# Clinicopathologic and prognostic features of breast cancer in young women: a series from North of Morocco

**DOI:** 10.1186/s12905-017-0456-1

**Published:** 2017-11-09

**Authors:** Joaira Bakkach, Mohamed Mansouri, Touria Derkaoui, Ali Loudiyi, Mohamed Fihri, Samia Hassani, Amina Barakat, Naima Ghailani Nourouti, Mohcine Bennani Mechita

**Affiliations:** 1 Human Genomic Research Laboratory, Faculty of Sciences and Techniques of Tangier, University Abdelmalek Essaâdi, Tangier, Morocco; 2 Oncology Clinic AL AMAL of Tangier, Tangier, Morocco; 3 Mathematics and Applications Laboratory, Faculty of Sciences and Techniques of Tangier, University Abdelmalek Essaâdi, Tangier, Morocco

**Keywords:** Breast cancer, Young women, BRCA1, BRCA2, Aggressive biology, Poor prognosis, Survival analysis

## Abstract

**Background:**

Literature data reported a higher frequency of breast cancer in young women (BCYW) in developing countries. BCYW is associated with delayed diagnosis, aggressive biology and poor prognosis. However, our knowledge of biological profile, treatment received and outcome of young patients is still limited in Morocco. We propose to analyze clinicopathologic, therapeutic and prognostic features of BCYW among a series of patients native and/or inhabitant of North of Morocco.

**Methods:**

We carried out a retro-prospective study of 331 infiltrating breast cancer cases registered between January 2010 and December 2015. Details of tumor pathology, treatment and outcome were collected. Disease-Free Survival (DFS) and Overall Survival (OS) were assessed by Kaplan-Meier analysis.

**Results:**

A total of 82 patients were diagnosed with breast cancer at the age of 40 or younger (24.8%). Median age was 36 years. More than one quarter (26%) of patients had family history of breast or ovarian cancer. Advanced stages accounted for 34.2% of cases. Median tumor diameter was 2.8 cm. Intermediate and high-grade tumors represented 47.6% and 40.2%, respectively. Nodal involvement was present in 58.5% and lymphovascular invasion was found in 47.7% of the patients. About two thirds (66.2%) of tumors were hormone receptor positive, 29.2% over-expressed HER2 receptor and 23% were triple negative. Patients underwent breast conserving surgery in 38.2% of cases, 61.7% were offered adjuvant chemotherapy and 84.6% received hormone therapy. Five-year DFS and OS were respectively 88.9% and 75.6%. Locoregional recurrence occurred in 2.8% of cases and 8.3% of patients developed distant metastases.

**Conclusion:**

Our findings are in accordance with previous studies that have shown a higher frequency of breast cancer among Moroccan young women. In line with literature data, clinicopathologic profile seems to be aggressive and prognosis is pejorative in our series.

## Background

Overall, breast cancer risk increases with the age. Incidence rates are shown to be lower among young women and the peak is within the age range of 65–74 years in more developed countries [1]. However in Morocco, the median age at onset for breast cancer is decade younger. According to the Greater Casablanca Cancer Registry report 2012, the peak incidence is at the age group 40–49 years [2]. Moreover, hospital-based series reported that breast cancer in young women (BCYW) do account for 8 to 25.4% [3–6], whereas this frequency does not exceed 7% in developed countries [7].

Young women deserve a particular attention because of the unique and complex challenges that these women are faced with [8]. These breast cancer patients have to deal with many issues related to fertility, self-image, psychosocial distress and professional integration. On the other hand, breast cancer in this age category tends to exhibit more aggressive features than that arising in older women [9–14], which makes the situation more difficult to handle.

BCYW is often associated with delayed diagnosis, aggressive biology, high relapse risk and poor survival [15–17]. The reason for this aggressiveness remains unclear. Historically, young age has been shown to be an independent predictor factor for poor prognosis [13, 18, 19]. However, recently with the emergence of molecular classification, this association with young age has disappeared in some studies [14, 20]. Currently, it is controversial whether BCYW is a unique biologic entity or it just reflects an over-representation of more aggressive molecular subtypes [21].

Young age for breast cancer occurrence often suggests an underlying genetic predisposition especially mutations in BRCA1/2 genes and less frequently in p53. Overall, BRCA1/2 genetic alterations may explain 10% of breast carcinomas arising in young women [22], but in studies from Arab countries much higher proportions have been reported [23, 24]. In Morocco, the young age for breast cancer diagnosis also strongly suggests that genetic factors could be involved.

We are conducting a genetic study to explore BRCA1 and BRCA2 mutations among breast cancer patients diagnosed at the age of 40 years or younger. This is the first genetic study including a case-series from North of Morocco. In this paper, we present a description of clinicopathologic, therapeutic and prognostic features among these south Mediterranean patients seen at the Oncology Clinic AL AMAL in Tangier, the only institution specialized in diagnosis and treatment of cancers available in the North Moroccan area at the beginning of the study.

## Methods

A total of 331 patients with invasive breast carcinoma were referred to the Oncology Clinic AL AMAL in Tangier between January 2010 and December 2015. Only patients diagnosed with breast cancer at the age of 40 or younger were recruited 82 (24.8%).

Medical records were reviewed and details of clinical characteristics, tumor pathology and treatment received were recorded. Family history, ethnic origin and any missing information were completed at follow-up visits. Patients with bilateral breast cancer were recorded only once.

Tumors were classified by means of TNM system 2010 and regrouped according to the American Joint Committee on Cancer stages (AJCC 2010). Histologic grading was performed according to SBR classification modified by Elston and Elliss (mSBR). Hormone receptors (HR, Estrogen ER and Progesterone PR) expression was evaluated based on percentage of tumor cells nuclear staining by the immunohistochemical method (IHC). The threshold for ER and PR positivity was ≥1%. HER2 (Human Epidermal growth factor Receptor 2) expression was processed immunohistochemicaly from paraffin-embedded cancer tissue using the HercepTest. Tumors scored 2^+^ were completed by FISH or CISH methods.

Patients were followed until 22 August 2016. The follow-up schedule was: every 4 months during the first 2 years, every 6 months for the next 2–5 years and every year thereafter. All patients who were not reviewed in the last visit were contacted again.

Five-year overall survival (OS) and Disease-free survival (DFS) were assessed using the Kaplan-Meier analysis. These were defined from the date of diagnosis to any tumor relapse or last follow-up (DFS), and to death or last follow-up (OS). The Log-Rank test was used to assess statistical difference between survival curves according to stages. This test was performed at a 5% level.

Participants provided an informed written consent. This study received approval from the Biomedical Research Ethics Committee in the Faculty of Medicine and Pharmacy in Rabat (CERB).

## Results

Among 331 patients diagnosed with breast cancer, 82 (24.8%) were at the age of 40 years or younger. The median age at diagnosis was 36 years (inferior age limit of 26). Most (98.8%) of the patients were Arabs or Amazighs (Berbers), whereas Europeans represent only 1.2% (Table 1).

**Table 1:** Demographic characteristics and family history (82 patients)

Parameter	Frequency
Age at diagnosis	
Median	36 years
≤ 35	45.1%
> 35	54.9%
Ethnicity	
Arab	68.3%
Amazigh	30.5%
European	1.2%
Family history	
Breast cancer in first- or second-degree relatives	22%
Breast cancer in third- or fourth-degree relatives	10%
Ovarian cancer	4%
Other cancers	42%
Absent	22%
Eisinger Score	
≤ 2	14.6%
3–4	72%
≥ 5	13.4%

More than one quarter (26%) of patients reported having at least one first- or second-degree relative with breast or ovarian cancer. Eisinger scoring system has revealed that 85.4% of patients were eligible for genetic counseling (Score value ≥3), of whom 15.7% had a score value more than 5 (Score value ≥5) showing thus an excellent indication for BRCA1/2 genetic testing (Table 1).

Combined mammography and ultrasound showed abnormalities in 97.5% of patients, of whom 72.5% had lesions that were highly suspicious of malignancy (ACR/BI-RADS category 5: American College of Radiology Breast Imaging-Reporting and Data System category 5). Only 2.5% were shown to be probably benign lesions (ACR 3) and were then reclassified during surveillance (Table 2). Clinically, 65.8% of patients were diagnosed with early-stage breast cancer, 22% locally advanced and 12.2% had metastatic disease. Of the whole series, distant visceral metastases were found in 14.6% of cases. Bone and brain metastases were seen in 9.8% and 2.4% respectively. Two patients had a personal history of contralateral breast cancer after a mean time interval of 118 months.

**Table 2:** Clinical characteristics (82 patients)

Parameter	Frequency
Presentation	
Breast mass	74.5%
Axillary adenopathy	6.4%
Pain	8.5%
Induration	6.4%
Nipple retraction	2.1%
Screen detected	2.1%
Imaging findings	
ACR3	2.5%
ACR4	26.8%
ACR5	70.7%
Positive diagnosis	
Biopsy	71.8%
Lympectomy	28.2%
Localization	
Right	38%
Left	62%
Multifocal disease	
Yes	13.5%
No	86.5%
Tumor size	
Median	2.8 cm
≤ 3	57.3%
> 3	42.7%

The histopathological characteristics are detailed in Table 3.

**Table 3:** Histopathological characteristics (82 patients)

Parameter	Frequency
AJCC stages	
Early	65.8%
Locally advanced	22%
Metastatic	12.2%
Histology	
Invasive carcinoma of No Special Type (NST)	95.2%
Lobular	2.4%
Mucinous	2.4%
mSBR grading	
I	12.2%
II	47.6%
III	40.2%
Lymph node status	
N-	41.5%
N+	58.5%
Lymphovascular invasion	
Present	47.7%
Absent	52.3%
Intraductal component	
Present	59.7%
Absent	40.3%
Hormone receptors	
Positive	66.2%
Negative	33.8%
HER2	
Positive	29.2%
Negative	70.8%
Molecular classification	
Luminal	66.2%
HER2	10.8%
Triple negative	23%

Patients with immediately operable (T1-T3N0N1) and immediately inoperable cancers (T4, N2-N3) represented 74.4% and 13.4% respectively. Those with metastatic disease at diagnosis accounted for 12.2% of cases. Of patients with immediately operable disease, 45.7% underwent breast-conserving surgery, whereas this proportion among those who had received neoadjuvant chemotherapy was only 10%.

Patients with HR-positive disease received hormone therapy in 84.6% of cases. Use of 5 years of Tamoxifen alone was recorded for 69.7% of patients. The Suppression of Ovarian Function in combination with Tamoxifen was indicated definitively by ovarian irradiation for 6.1% of cases or reversely by means of Luteinizing Hormone-Releasing Hormone (LH-RH) agonists (15.1%). Patients who became definitively postmenopausal by the time of treatment after 2 or 3 years of Tamoxifen were switched to an Aromatase Inhibitor for the 2 or 3 years thereafter (9.1%).

More information about treatment received is presented in Table 4.

**Table 4:** Treatment modalities (82 patients)

Parameter	Frequency
Surgery	
No	16.7%
Yes	83.3%
Radical	61.8%
Conservative	38.2%
Chemotherapy	
No	9.1%
Yes	90.9%
Neoadjuvant	25%
Adjuvant	61.7%
Palliative	13.3%
Radiotherapy	
Yes	73.4%
No	26.6%
Hormonotherapy (HR+)	
Yes	84.6%
No	15.4%

**Fig. 1:**
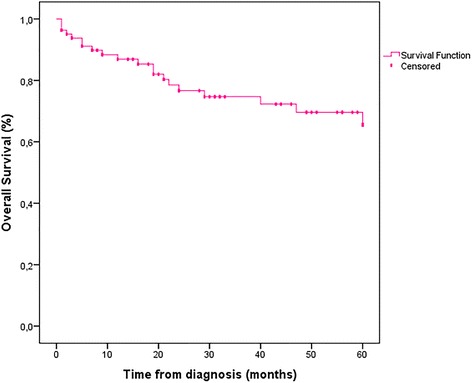
Overall survival at 5 years for 82 patients

**Fig. 2:**
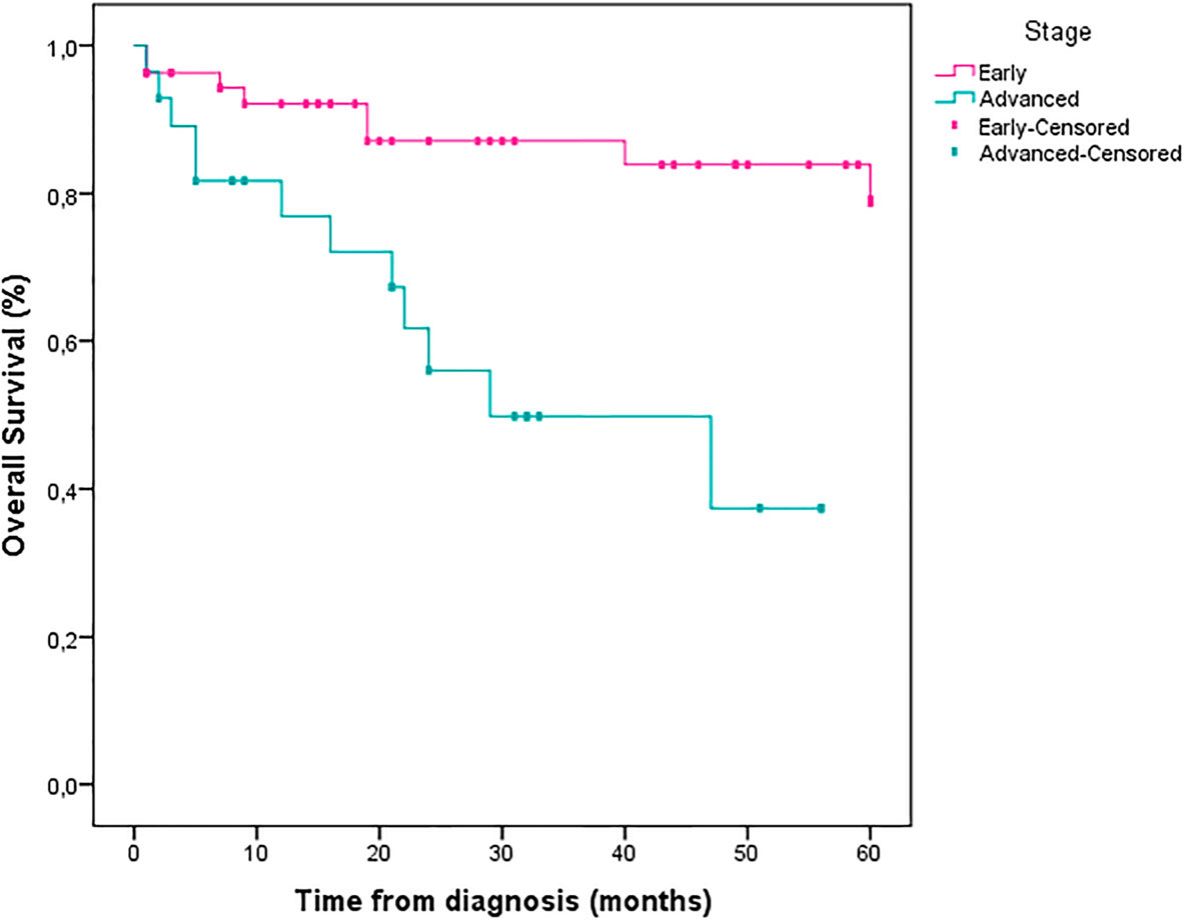
Survival for 82 patients according to AJCC stages, *pvalue* = 0.001

**Fig. 3:**
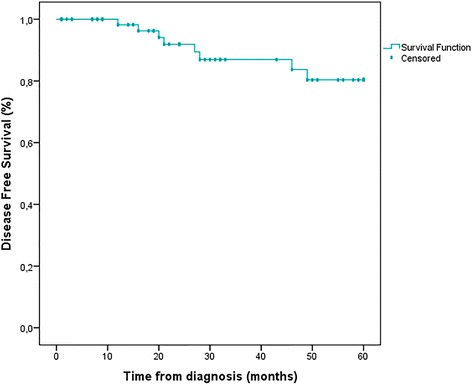
Disease-free survival at 5 years for non-metastatic patients

During the total period of follow-up, 2.8% of patients developed isolated locoregional recurrence and 8.3% had distant metastases after a mean duration of 27.4 months (12–49 months). The estimated DFS at 5 years for non-metastatic patients was 88.9% and OS for the whole case-series was 75.6%. Early-stage breast cancer patients had superior OS at 5 years compared to advanced breast cancer patients (85.2% Vs. 57.1%, *pvalue* = 0.001 < 0.05) (Table 3, Figs. 1, 2 and 3).

## Discussion

BCYW is a complex topic to discuss because there is no unified definition of “young age”. The most used definitions place the limit at 35 or 40 years. This definition’s variability creates non-homogeneity between studies, which complicates the interpretation of the results. In the present work we have chosen to define early onset breast cancer when diagnosed at the age of 40 or younger.

Breast cancer is described as young women disease in Arab world and other developing nations like some African and Asian countries [25, 26]. Compared to that observed in western countries, median age for breast cancer diagnosis is a decade younger, and approximately two thirds of patients are aged under 50 [27]. Our results are in accordance with previous studies describing a higher proportion of early onset breast cancer in Morocco. Our frequency among women diagnosed at the age of 40 or younger (24.8%) is consistent with data from Greater Casablanca Cancer Registry (22%), the only population-based registry that exists in Morocco since 2007 [28]. This result is also similar to a recent study conducted at the National Institute of Oncology in Rabat (24.9%) [6]. Other national hospital-based series have showed disparate results among women aged under 36 [3–5]. In our study, women aged under 36 represent 11.2%. As our case-series is limited, we could not draw any conclusions about the frequency of early onset breast cancer in the North Moroccan region.

The young age for breast cancer diagnosis in Morocco raises the question of whether demographic, genetic or environmental factors are implicated. Young age pyramid has been invoked to explain the higher frequency compared to western countries. Moreover, absence of mass screening could be an additional factor that suggests an under-estimation of breast cancer among older women [29, 30]. This factor, alone, is however insufficient to explain the young age for breast cancer occurrence in Morocco. The genetic component should be more explored, since genetic studies including young women are very scarce nationwide, and there is still insufficient data to provide conclusive evidence [31, 32]. In our study, the higher frequency could be explained by a recruitment bias or could simply be due to the young age of the population in this region [33].

In the absence of a national registry that covers the whole Moroccan population, the real frequency of early onset breast cancer remains to be determined. Larger studies and subsequent analyses are extremely warranted.

Morocco is an ethnically diverse North African country that comprises Arabs, Amazighs, Sub-Saharan groups, Jews and Europeans. In our series including patients native and/or inhabitant of North of Morocco, most (98.8%) patients were Arab or Amazigh descent. These are the most frequent ethnicities in this region. Our case-series could not provide any conclusion about the contribution of these ethnic groups in BCYW. To the best of our knowledge, no data is available at the national scale about the incidence of BCYW among these different ethnic groups.

Presence of a familial background has been identified as an important risk factor for developing breast cancer at an early age [34], and to be suggestive of a hereditary syndrome. In our study, 26% of patients had positive family history of breast or ovarian cancer, in accordance with previously reported data from Sidoni et al. [9] study (24%). Larger series have reported much higher proportions up to 48% [12, 16, 17, 35].

It has been recommended that young women with personal history of breast cancer should be considered for genetic counseling, and BRCA1/2 testing even when not meeting familial criteria. Published data about referral patterns showed that the proportion of young breast cancer patients who underwent BRCA1/2 testing has been increased in the last decade particularly between 2012 and 2013 [36]. This trend of genetic counseling referrals observed in developed countries has been related to the media attention to the hereditary breast cancer risk “The Angelina Jolie Effect” [37, 38]. Sadly, such data is yet to become available nationwide because the interest for genetic disorders is recent in Morocco, and no medical genetic centers have been established until the end of 1990s [39].

The age cut-off used for BRCA1/2 genetic screening among young women varies widely between countries. French recommendations use a cut-off of 36 years [40], whereas the National Comprehensive Cancer Network guidelines recommend screening in women aged younger than 45 [41]. Adoption of any of these international standards should generally be tailored to each population’s specificity. Decision-making is however difficult for Morocco as well as other similar countries, because no legal framework for genetic counseling services is available, besides the knowledge about mutation prevalence is still limited to provide sufficient evidence. Further genetic studies would be therefore extremely helpful in order to define the appropriate age cut-off.

Young age at onset for breast cancer has been shown to increase the risk of subsequent contralateral cancer. This higher risk might be explained somewhat by genetic susceptibility especially mutations in BRCA1 and BRCA2 genes [42]. In our series, two patients had a personal history of contralateral breast cancer. The first breast cancers were of luminal type, whereas the second cancers were triple negative. Such difference was not observed by Huo et al. [43] who described a strong concordance in hormone receptor status between primary and second breast cancers.

Diagnosis in young women has been considered difficult to establish both clinically and mammographically due to the increased breast density within this age group. Generally, this is translated into more advanced stages at disease presentation. In line with these observations, we described a diagnosis delay, but the proportion of advanced-stage disease remains much higher than that reported in western series (34.2% Vs. 19%) [16].

It has been reported that breast cancer occurring in younger women has more aggressive features. The tumors were more likely to be undifferentiated, with higher proliferation index, more nodal involvement, less positive hormone receptor status and with an over-expression of HER2 [9–14].

Proportion of high-grade tumors in our series (40.2%) remains relatively low compared with western series, which reported more higher rates up to 58.9% among young women under 40 [11, 12, 15–17]. This could be explained by the presence in our study of an important rate of intermediate grade tumors (47.6%).

Having node involvement (≥ 4 nodes) is a poor prognostic factor which is considered as an indication for adjuvant chemotherapy according to the 2015 St Gallen International Consensus guidelines [44]. In previous publications, lymph node-positive disease in young women under 40 years seems to be ranged from 39.2% to 53% [9, 11, 12, 15, 17]. In the current study, 58.5% of patients had positive lymph nodes.

Several studies have shown age-specific disparity in hormone receptors status [9–12, 14]. In recent series, rate of hormone-negative tumors has been around 34% [15–17, 45]. The proportion of HER2 over-expressing tumors in published reports ranged from 15.2% to 48% [9, 15–17, 45]. In accordance with these data, we found that 33.8% of tumors were HR-negative and 29.2% over-expressed HER2.

Tumor biology and molecular pathways involved in early-onset breast cancers are widely unknown. Based on IHC or gene expression profiling, published data have shown that for unclear reasons younger women have tendency for more aggressive subtypes [10, 14, 16, 45]. The proportion of luminal cancers in our study is almost identical (66.2%) to that reported by Collins et al. [16], but we have not sufficient data for Ki-67 to permit classification into types A and B. HER2 and triple negative phenotypes were found respectively in 10.8% and 23% of the cases, consistent with Keegan et al. [45] study. Clinicopathologic and prognostic features of the triple negative group diagnosed in our institution have been recently published. This study revealed that approximately two thirds (63.6%) of the patients were non-menopausal [46].

The choice of surgical treatment type depends mainly on risk factors of local recurrence, ratio of cosmetic/carcinological results and patient’s informed decision. Young age (especially ≤35 years) has been considered as risk factor for local recurrence following breast-conserving surgery [47], but despite this increased risk there is a great evidence for no survival benefit for undergoing radical treatment rather than conserving surgery [47, 48].

We described in our study that most (61.8%) of patients underwent radical surgery. This could be partly explained by the presence of many limitations for management of breast conserving surgery. The radiological surveillance by Magnetic Resonance Imaging (MRI) is often discussed for BCYW, as it is the most sensitive test (more than 90%) for detecting small and/or multicentric relapse in dense breasts [49, 50], but unfortunately this imaging modality is still expensive, which substantially hinders better management.

Several publications have revealed that breast cancer young patients have had poor prognosis with increased risk of locoregional recurrence and lower survival [11–13, 18, 19, 51]. Buckley et al. [52] reported a 5-year locoregional recurrence rate of 5.56%, whereas another study by Plichta et al. [53] described a 5-year locoregional recurrence rate of 2%, almost similar to that found in our series (2.8%).

Data from the present study showed that the 5-year OS (75.6%) was comparable to that previously reported in older series [19, 51], but this was still lower compared with recent reports [17, 53]. Furthermore, this OS was much lower for patients with advanced-stage disease. Survival according to stages revealed that OS at 5 years in advanced stages was significantly inferior to that in early stages (57.1% Vs. 85.2%, *pvalue* = 0.001 < 0.05). Overall, the poor prognosis observed in our series may be partly explained by a substantial delayed diagnosis, but other factors are likely to be involved including the aggressive biology or potential socioeconomic factors.

The present study showed some limitations. This is a size limited case-series recruiting patients from only one health institution. As such, it could not be representative of the whole North Moroccan region. The retrospective character of some data represents another limitation. In addition, our study is conducted in the private sector, which could represent a recruitment bias.

## Conclusion

Our results are in accordance with literature data reporting higher proportion of breast cancer among young Moroccan women. Larger studies are warranted to confirm our findings.

Interestingly, our ongoing genetic study for identifying germline mutations in BRCA genes would contribute to a better understanding of the genetic profile of patients involved in this series.

Consistent with published reports, we described aggressive biologic features. There was seen much higher proportion of advanced-stage disease in our study, which reflects somewhat a substantial delay in diagnosis. In terms of treatment choices, our patients underwent radical mastectomy rather than conserving surgery, which adds further challenge for these young women at the emotional and psychosocial levels, especially in the absence of a specialized psychological support.

## Abbreviations

ACR: American College of Radiology
AJCC: American Joint Committee of Cancer
BCYW: Breast cancer in young women
BI-RADS: Breast imaging-reporting and data system
CI: Confidence interval
CISH: Chromogenic in-situ hybridization
DFS: Disease-free survival
ER: Estrogen receptor
FISH: Fluorescence in situ hybridization
HER2: Human Epidermal growth factor Receptor 2
HR: Hormone Receptors
IHC: Immunohistochemistry
MRI: Magnetic resonance imaging
mSBR: Scarf-Bloom-Richardson classification modified by Elston and Elliss
NST: No special type
OS: Overall survival
PR: Progesterone receptor
